# Investigating the Effect of *Ferula assafoetida* L. Extract and *Farnesiferol* on Adapter Activation and Inhibition of Metastasis of Genes Involved in the Severity of Malignancy in Breast Cancer Cell Lines

**DOI:** 10.1002/fsn3.70686

**Published:** 2025-07-26

**Authors:** Farinaz Malakotikhah, Kahin Shahanipour, Ramesh Monajemi, Ali Mohammad Ahadi, Ali Asghar Rastegari

**Affiliations:** ^1^ Department of Biochemistry, Fal.C. Islamic Azad University Isfahan Iran; ^2^ Department of Biology, Fal.C. Islamic Azad University Isfahan Iran; ^3^ Departmant of Genetics, Faculty of Science Shahrekord University Shahrekord Iran

**Keywords:** breast cancer, *Farnesiferol*, *Ferula assafoetida* L., target therapy

## Abstract

The discovery of efficient therapies for metastatic illness is essential since breast cancer continues to pose a serious health threat. The purpose of this study was to examine the possibility that extract from *Ferula assafoetida* L. and a natural substance called *farnesiferol* may stop the spread of breast cancer by preventing the expression of genes linked to cell invasion. *Ferula assafoetida* L. extract and *farnesiferol* were tested for cytotoxicity using the MTT assay after MCF‐7 and MDA‐MB‐231 cells were punctured. Apoptosis induction was assessed by flow cytometry. The expression of apoptosis‐related genes was evaluated utilizing quantitative real‐time PCR. The evaluation of Total Antioxidant Capacity (TAC) was performed. A study found that *farnesiferol* significantly increased the survival rates of MCF‐7 and MDA‐MB‐231 cells after 72 h. The IC_50_ of *Ferula assafoetida* L. and *farnesiferol* was 1000 and 500 μg/mL, respectively. Treatment with *Ferula assafoetida* L. and *farnesiferol* led to apoptosis in MCF‐7 cells at 41.1% and 62.7%, respectively. In MDA‐MB‐231 cells treated with *Ferula assafoetida* L. and *farnesiferol*, apoptosis rates were around 1.60% and 2.27%. The transcription levels of *GPX*, *P53*, and *MMP2* genes were also significantly increased in both groups. *Farnesiferol* has promise as a powerful therapeutic agent for breast cancer by modulating cellular proliferation and apoptosis. Additional study is necessary to confirm the therapeutic effectiveness of *farnesiferol* and elucidate its mechanisms in breast cancer.

## Graphical abstract

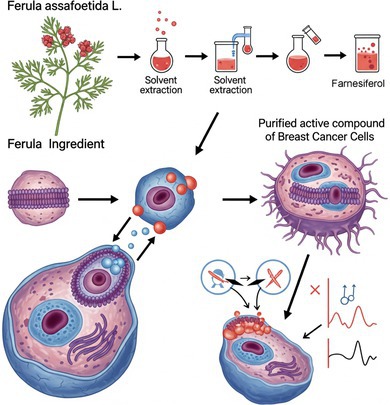

## 1 Introduction

Cancer remains a significant global health challenge, characterized by uncontrolled cell growth and the ability of these abnormal cells to invade and spread throughout the body (Sung et al. 2021). The rising incidence of cancer places a substantial burden on public health systems. While treatments like surgery, chemotherapy, radiation, immunotherapy, and targeted therapies exist, significant hurdles persist, including drug resistance, severe side effects, and the high cost of treatment (Siegel et al. 2022; Bray et al. 2018).

Breast cancer, a prevalent malignancy in women, originates from the malignant transformation of breast tissue cells. It encompasses diverse subtypes with varying biochemical and clinical features influenced by factors such as hormone receptor status, HER2 expression, and genetic abnormalities (Bray et al. 2018; Harbeck and Gnant 2017). Current breast cancer treatments often come with considerable side effects like fatigue, nausea, hair loss, and an increased risk of secondary cancers (DeVita et al. 2019). Furthermore, the development of drug resistance limits the long‐term effectiveness of many therapies (Longley and Johnston 2005). These challenges, coupled with the exorbitant cost of treatment, have spurred interest in alternative and complementary approaches, particularly herbal targeted therapy. Certain phytochemicals demonstrate promising anti‐cancer properties, including the capacity to inhibit tumor growth, induce apoptosis, and modulate immune responses (Bishayee et al. 2022). Green tea, *Ferula assafoetida* L., turmeric, and ginger contain compounds that have shown promise in preclinical and early clinical research for breast cancer treatment. However, thorough scientific investigation is crucial to fully ascertain their efficacy, safety, and appropriate dosage (Longley and Johnston 2005; Bishayee et al. 2022; Aggarwal et al. 2018).

*Ferula assafoetida* L., commonly known as asafoetida, is a perennial plant with a rich history in traditional medicine (Mahendra and Bisht 2012). Recent research has highlighted its potential anti‐cancer capabilities. Studies suggest that constituents of asafoetida, including sulfur‐containing compounds, flavonoids, and terpenes, may possess anti‐proliferative properties against various cancer cell lines (Khan et al. 2017). These compounds may induce apoptosis in cancer cells, reduce tumor development, and decrease metastasis. While promising, further research is needed to comprehensively elucidate the mechanisms and clinical utility of asafoetida in cancer therapy (Ullah et al. 2019).

Asafoetida contains a variety of anti‐cancer compounds. These include sulfur‐containing chemicals like disulfides and trisulfides, which have demonstrated anti‐proliferative effects on cancer cells (Saleem et al. 2004). Additionally, asafoetida is rich in flavonoids, a class of plant pigments with antioxidant and anti‐inflammatory properties, some of which have exhibited anti‐cancer effects. Terpenes, another significant category of chemical molecules found in plants, are also present in asafoetida (Kashiwada et al. 1990). Farnesiferol, a sesquiterpene alcohol, has garnered considerable attention for its potential anti‐cancer capabilities. Farnesiferol has shown the ability to suppress tumor proliferation and induce apoptosis in several cancer cell lines (Lee et al. 2011).

Utilizing the isolated active component farnesiferol for breast cancer treatment, rather than the whole plant extract, offers several potential benefits (Lee et al. 2011). Firstly, it allows for precise dosage and enhanced regulation of the active chemical delivered to the body, thereby reducing potential negative effects from other plant constituents. Secondly, it streamlines drug development and manufacturing procedures, as isolating the active component improves the consistency and purity of the medicinal substance (Raskin et al. 2002). Thirdly, using isolated farnesiferol may enhance its bioavailability and effectiveness compared to the whole plant extract, which might contain components that could hinder its absorption or function (Pan et al. 2013).

The investigation of farnesiferol‐based targeted plant therapy for breast cancer is critically significant for several reasons. Initially, it presents a prospective pathway for the development of innovative and more effective therapies for this debilitating condition (Pan et al. 2013; Cragg and Pezzuto 2016). Conventional chemotherapies often have significant adverse effects, whereas targeted treatments are designed to selectively target cancer cells, thereby reducing damage to healthy organs. By elucidating the interaction of farnesiferol with specific molecular targets in breast cancer cells, researchers may formulate more targeted and individualized therapy approaches (Cragg and Pezzuto 2016).

Secondly, investigating plant‐derived medicines such as farnesiferol aligns with the increasing enthusiasm for natural and integrative medicine. Many individuals seek alternative or complementary therapies, and phytochemicals offer a promising avenue for innovative medicines that may exhibit fewer adverse effects compared to synthetic medications (Li et al. 2019). Moreover, exploring the mechanisms of action of farnesiferol may provide significant insights into the intricate biology of breast cancer, potentially facilitating the identification of novel therapeutic targets and the advancement of more efficacious conventional treatments (Newman and Cragg 2016).

Extensive research has been conducted on the effects of *Ferula assafoetida* L. extract and farnesiferol on various cancer cell lines. Studies have demonstrated the anti‐proliferative and apoptotic effects of *Ferula assafoetida* L. extract on a range of cancer cells, including those from colon, liver, and lung cancers, in addition to breast cancer. These studies often highlight the role of sulfur‐containing compounds and other phytochemicals present in the extract in mediating these effects.

More specifically, farnesiferol has been rigorously investigated for its targeted anti‐cancer properties. Research has shown its ability to inhibit proliferation and induce apoptosis in various breast cancer cell lines (e.g., MCF‐7, MDA‐MB‐231), often through mechanisms involving cell cycle arrest and modulation of key signaling pathways like MAPK and PI3K/Akt. Beyond breast cancer, farnesiferol has also exhibited promising results in studies involving other cancer types, such as ovarian cancer and leukemia, by interfering with cell survival and proliferation. These studies provide a strong foundation for further exploration of farnesiferol as a potential therapeutic agent.

While previous studies have broadly demonstrated farnesiferol's anti‐proliferative and pro‐apoptotic effects across various cancer cell lines, the precise molecular mechanisms by which farnesiferol selectively targets breast cancer cells, particularly specific subtypes, remain largely uncharacterized. This research aims to address this critical gap by elucidating the specific signaling pathways (e.g., cell cycle regulatory proteins, specific apoptotic cascades, or interactions with key oncogenes/tumor suppressors) modulated by farnesiferol in a relevant breast cancer cell line. By unraveling these intricate mechanisms, this study seeks to provide a deeper understanding of farnesiferol's therapeutic potential and inform the rational design of targeted therapies for breast cancer. The results of this in vitro investigation, evaluating the effects of farnesiferol on cell viability, apoptosis, and cell cycle progression, will contribute novel insights into its mode of action, potentially aiding in the creation of innovative and more effective treatments for breast cancer.

## 2 Materials and Methods

### 2.1 Materials and Chemicals

The MCF‐7 and MDA‐MB‐231 breast cancer cell lines were purchased from the Iranian Pasteur Institute's national cell collection (Pasteur, Iran). All requisite cell culture reagents, comprising trypsin–EDTA (0.25%), fetal bovine serum (FBS), Dulbecco's Modified Eagle Medium (DMEM), buffers, and a mixture of penicillin, streptomycin, and amphotericin B as antibiotics, were procured from ThermoFisher Scientific (India). Furthermore, the MTT Assay Kit was procured from Bio‐Idea (Iran). We purchased sterile culture flasks and multi‐well plates from ZistYarSanat Ltd. (ZistYarSanat, Iran). *Farnesiferol* (Molecular Weight: 382.49, Purity: > 99%) was supplied by TargetMol Company Inc. (Boston, USA). DMSO was supplied by ZistYarSanat Ltd. (ZistYarSanat, Iran; https://zys‐group.ir).

### 2.2 Preparation of *Ferula assafoetida* L. Extract

The Agricultural and Natural Resources Research Centre in Isfahan Province, Iran, produced the gum of *Ferula assafoetida* L. To manufacture the methanolic extract of *Ferula assafoetida* L., 50 g of ground *Ferula assafoetida* L. gum was dissolved in 20 g of 100% methanol and stirred for 24 h in darkness using a heated stirrer. The resultant solution was further filtered through a 0.22‐μm filter and dried in an oven at 70°C.

### 2.3 Preparation of Stock Solutions

After dissolving 1 mg of powdered *Ferula assafoetida* L. and 1 mg of powdered *Farnesiferol* in 1 mL of DMSO, the mixture was shaken for 24 h at 37°C and 180 rpm. The current solution was maintained at 4°C and designated as a *Ferula assafoetida* L. and *Farnesiferol* stock solution. The total amount of the stock solutions was 1 mg/mL (1000 μg/mL).

### 2.4 Cell Culture

MCF‐7 and MDA‐MB‐231 were acquired from the Pasteur Institute of Iran in Tehran. The cells were cultured in full DMEM media supplemented with 10% FBS, 100 units/mL penicillin, and 100 μg/mL streptomycin and cultured at 37°C in a 5% CO_2_ atmosphere. The culture media were changed biweekly until the cells attained 70%–80% confluence. The cells were collected by immersing them in a 0.25% trypsin–EDTA solution (DACell, Iran). Following centrifugation, the cells were enumerated and suspended in PBS (Gibco, USA) for future investigation.

### 2.5 In Vitro Cytotoxicity Investigation

The half‐maximal inhibitory concentration (IC_50_) was computed utilizing the MTT technique to assess the inhibitory impact of *Farnesiferol* and *Ferula assafoetida* L. on breast cancer cells. IC_50_ is the concentration of a pharmaceutical agent that reduces the proliferation of neoplastic cells by 50%. It is frequently employed to assess the therapeutic efficacy of a therapy in the laboratory. Each well of 96‐well plates contained 5 × 10^6^ cells, which were treated with PBS, *Ferula assafoetida* L., and *Farnesiferol* at doses of 1000, 500, 250, 125, and 62.5 μg/mL for durations of 24, 48, and 72 h. Upon completion of the incubation period, the culture media was extracted. Subsequently, 100 μL of MTT (Sigma‐Aldrich, USA) reagent (0.5 mg/mL in PBS) was introduced to each well, and the plate underwent incubation at 37°C for 3 h. The MTT solution was removed, and 100 μL of DMSO (Sigma‐Aldrich, USA) was incorporated to solubilize the formazan crystals. The wavelength of the absorbance of each sample was quantified at an optical density (OD) of 570 nm employing an ELISA reader (ChroMate, The Netherlands). Cell proliferation was measured using the equation below.

$$\text{Cell proliferation}\;\left(\%\right)=\frac{{\mathrm{OD}}_{T}}{{\mathrm{OD}}_{C}}\times 100$$

OD_C_ refers to the optical density of untreated cells, while OD_T_ refers to the optical density of treated cells. Cell survival results were analyzed, and IC_50_ values were measured based on the dose–response pattern.

### 2.6 Flow Cytometry and Cell Apoptosis Measurements

Cell apoptosis study was conducted utilizing an Annexin V‐FITC apoptosis identification kit from BD Biosciences (Franklin Lakes, NJ, USA). Cells were seeded in 6‐well plates at a density of 5 × 10^6^ cells per well. Subsequently, they were administered treatment for 72 h with the IC_50_ dosage of PBS, *Ferula assafoetida* L., and *Farnesiferol*. Following two rinses with cold, sterile PBS (pH 7.4), a solution consisting of 5 × 10^6^ cells per well was formulated on a 6‐well plate employing the 1X binding buffer supplied by the reagent. Cells were treated following the manufacturer's guidelines by including exact quantities of Annexin V‐FITC (green fluorescence) and propidium iodide (red fluorescence). The incubation period was 10 min at standard room temperature. The cell samples were transferred to a flow cytometric tube and then analyzed using flow cytometry (Spectrum Two, Perkin Elmer, USA) in the final stage phase.

### 2.7 Investigation of Cell Cycle Arrest

Propidium iodide staining (PI) was used to measure cell growth. The DNA content is employed to ascertain the stage of the cell cycle since the quantity of DNA present is directly proportional to the binding of propidium iodide to the DNA. Cells were grown in complete media in 6‐well plates at a count rate of 5 × 10^6 cells per well. Following the overnight incubation and three washes with PBS, cells were exposed to PBS, *Ferula assafoetida* L., and *Farnesiferol* in full medium for 72 h. Cells were then collected and preserved in 70% cooled ethanol at 4°C overnight. Cells were then subjected to 450 μL of PI mixture (including RNase) in the absence of light for 20 min at ambient temperature. The cells were then analyzed using flow cytometry. Three replications of the tests were conducted.

### 2.8 Real‐Time PCR

The IC_50_ concentration of *Ferula assafoetida* L. and *Farnesiferol* was determined. MCF‐7 and MDA‐MB‐231 cells were treated for 24, 48, and 72 h at the IC_50_ dosage of *Ferula assafoetida* L. and *Farnesiferol*. Real‐time polymerase chain reaction (PCR) was employed to measure the gene expression of the *MMP2*, *GPX*, and *P53* genes. The research was conducted at IC_50_ doses of *Ferula assafoetida* L. and *Farnesiferol*. *ActinB* served as the reference gene. Total RNA was extracted employing the TRIzol protocol (Thermo Fisher Scientific, Waltham, MA, USA), and the production of cDNA was conducted employing a cDNA synthesis kit (Parstous, Iran). The thermal protocol for Real‐time PCR (Ausdiagnostic, Australia) included an initial phase at 95°C for 5 min, followed by 35 cycles of 95°C for 30 s, 60°C for 30 s, and 72°C for 30 s. Real‐time PCR was conducted employing 7.5 μL of SYBR‐PCR master mix (Amplicon, Denmark), with each primer at an amount of 100 nM and 1 μL of RT product, adjusted to a final volume of 15 μL with water. The primer sequences for the specified genes are presented individually for the forward and reverse in Table 1. The $2^{–\Delta \Delta C_{T}}$ technique was used to ascertain the fold changes in the control group. The experiments were conducted at three distinct times.

**TABLE 1:** List of primers utilized in this study.

Gene	Sequence (5′ → 3′)	Accession number	Size (bp)	References
*GPX*	F: 5′‐CATGCAATCAGTTCGGACAC‐3′ R: 5′‐TCACCATTCACTTCGCACTTC‐3′	NM_008160.6	130	This study
*P53*	F: 5′‐CATGACGGAGGTTGTGAGGC‐3′ R: 5′‐GGTTCTGTCATCCAAATACTC‐3′	MG595980.1	127	This study
*MMP2*	F: 5′‐CAACTACAACTTCTTCCCTCGC‐3′ R: 5′‐GCTCGGGCCTTAAAAGTATG‐3′	NM_008610.3	129	This study
*ActinB*	F: 5′‐CACCCGCCGCCAGCTCACC‐3′ R: 5′‐CACGATGGAGGGGAAGACGG‐3′	NM_001101.5	124	This study

### 2.9 Total Antioxidant Capacity (TAC) Measurement

This method employs a laboratory radical that oxidizes colorless ABTS, producing green‐blue ABTS˙+. The existence of antioxidant compounds in the sample regenerates ABTS˙+, leading to the restoration of colorless ABTS. The absorption value of the samples at 420 nm, together with the peak wavelengths of 660 and 740 nm, is quantified by ELISA after 5 min. A standard curve is generated using the standard absorption values of Trolox. The total antioxidant content of the samples is calculated using the standard curve.

### 2.10 Statistical Analysis

Statistical analysis was conducted using GraphPad Prism 4.0, presenting data as mean ± standard deviation and mean ± standard error of the mean (SEM). The data were analyzed with a one‐way analysis of variance (ANOVA). A *t*‐test with a *p*‐value of 0.05 was used to compare group pairings.

## 3 Results

### 3.1 Assessment of Cell Cytotoxicity

This research assessed the cytotoxicity of *Ferula assafoetida* L. and *Farnesiferol*, along with the determination of IC50, utilizing the MTT test. MCF‐7 and MDA‐MB‐231 cells were subjected to various doses (1000, 500, 250, 125, and 62.5 μg/mL) of *Ferula assafoetida* L. and *Farnesiferol* for 24 h.

**FIGURE 1:**
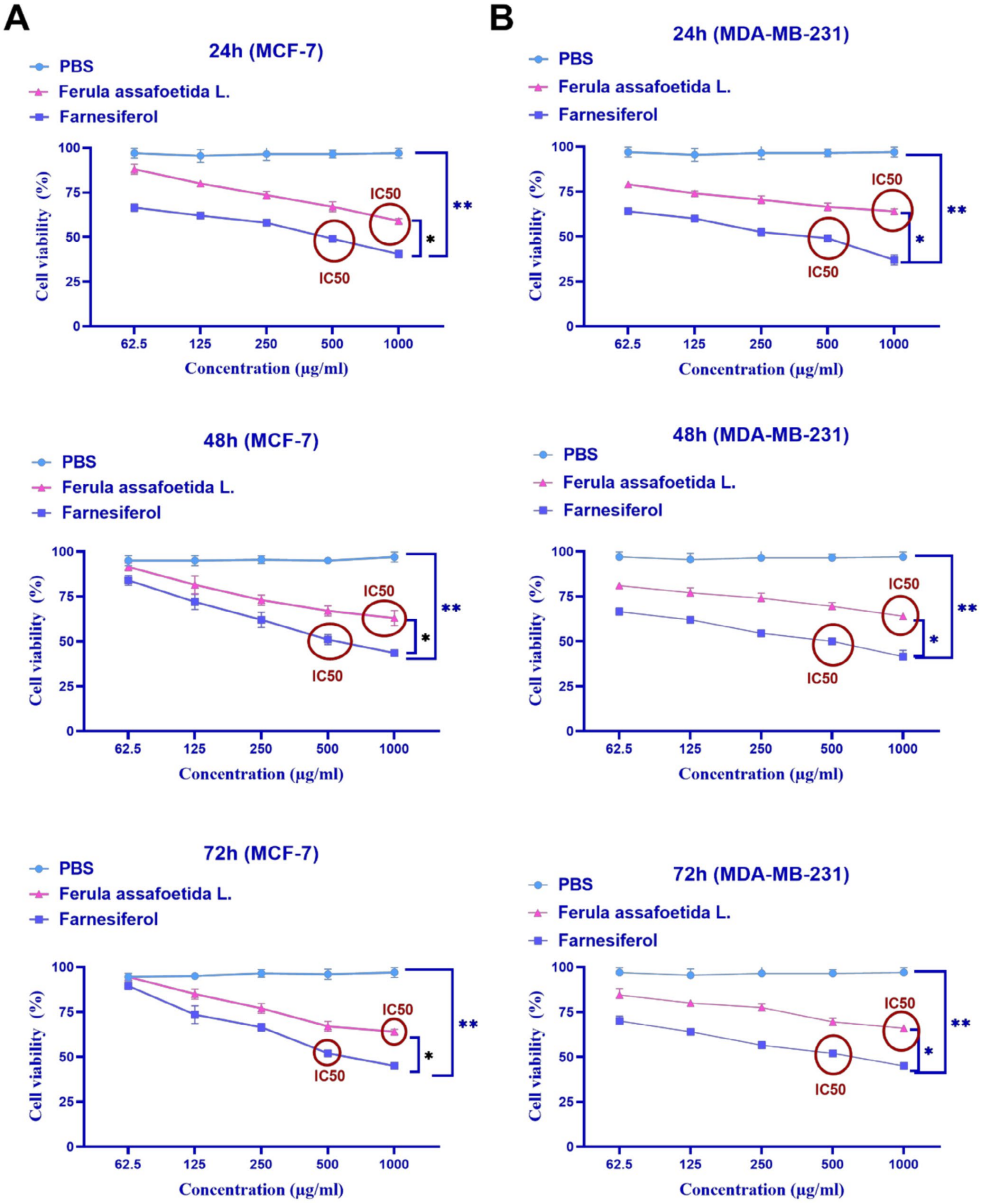
Effect of varying concentrations of Ferula assafoetida L. extract and farnesiferol on the viability of (A) MCF‐7 and (B) MDA‐MB‐231 breast cancer cells after 24, 48, and 72 h of treatment. Cell viability was assessed using the MTT assay and compared to the PBS‐treated control group. Data are presented as mean ± SD. *p < 0.05; **p < 0.01 indicate statistically significant differences compared to control.

This study's findings indicated that treating the MCF‐7 cell line with *Farnesiferol* at a dose of 500 μg/mL led to the mortality of 50% of the cells. The IC50 for this investigation was 500 μg/mL (Figure 1). Following treatment with *Farnesiferol* for 24, 48, and 72 h, MCF‐7 cells exhibited survival rates of 49%, 51%, and 52% at a dosage of 500 μg/mL. The treatment of the MCF‐7 cell line with *Farnesiferol* at 24, 48, and 72 h did not exhibit a significant change. The treatment of the MCF‐7 cell line with varying dosages of *Ferula assafoetida* L. exhibited considerable cytotoxicity at a concentration of 1000 μg/mL. Following treatment with *Ferula assafoetida* L. for 24, 48, and 72 h, MCF‐7 cells exhibited survival rates of 59%, 63%, and 64% at a dosage of 1000 μg/mL. The administration of the MCF‐7 cell line with *Ferula assafoetida* L. at 24, 48, and 72 h did not exhibit a significant change.

This research revealed that *Farnesiferol* has cytotoxic properties against the MDA‐MB‐231 cell line. Treatment with 500 μg/mL *Farnesiferol* induced 50% cell mortality, hence determining the IC50 value for this chemical. *Farnesiferol* exhibited cytotoxicity at a dose of 500 μg/mL, resulting in cell survival rates of 49%, 50%, and 52% after 24, 48, and 72 h of treatment, respectively. No notable variations in cell viability were seen when MDA‐MB‐231 cells were exposed to *Farnesiferol* for 24, 48, and 72 h. Likewise, *Ferula assafoetida* L. extract exhibited cytotoxicity at a concentration of 1000 μg/mL, resulting in cell survival rates of 64%, 64%, and 66% after 24, 48, and 72 h of treatment, respectively. No notable changes in MDA‐MB‐231 cell viability were seen throughout the various periods for *Ferula assafoetida* L. treatment (Figure 1B). Also, the growth graph of the effect of different doses of *Ferula assafoetida* L. and *Farnesiferol* on the viability of MCF‐7 and MDA‐MB‐231 cells after 24, 48, and 72 h compared to the PBS control group is shown in Figure 1.

### 3.2 Evaluation of Apoptosis

**FIGURE 2:**
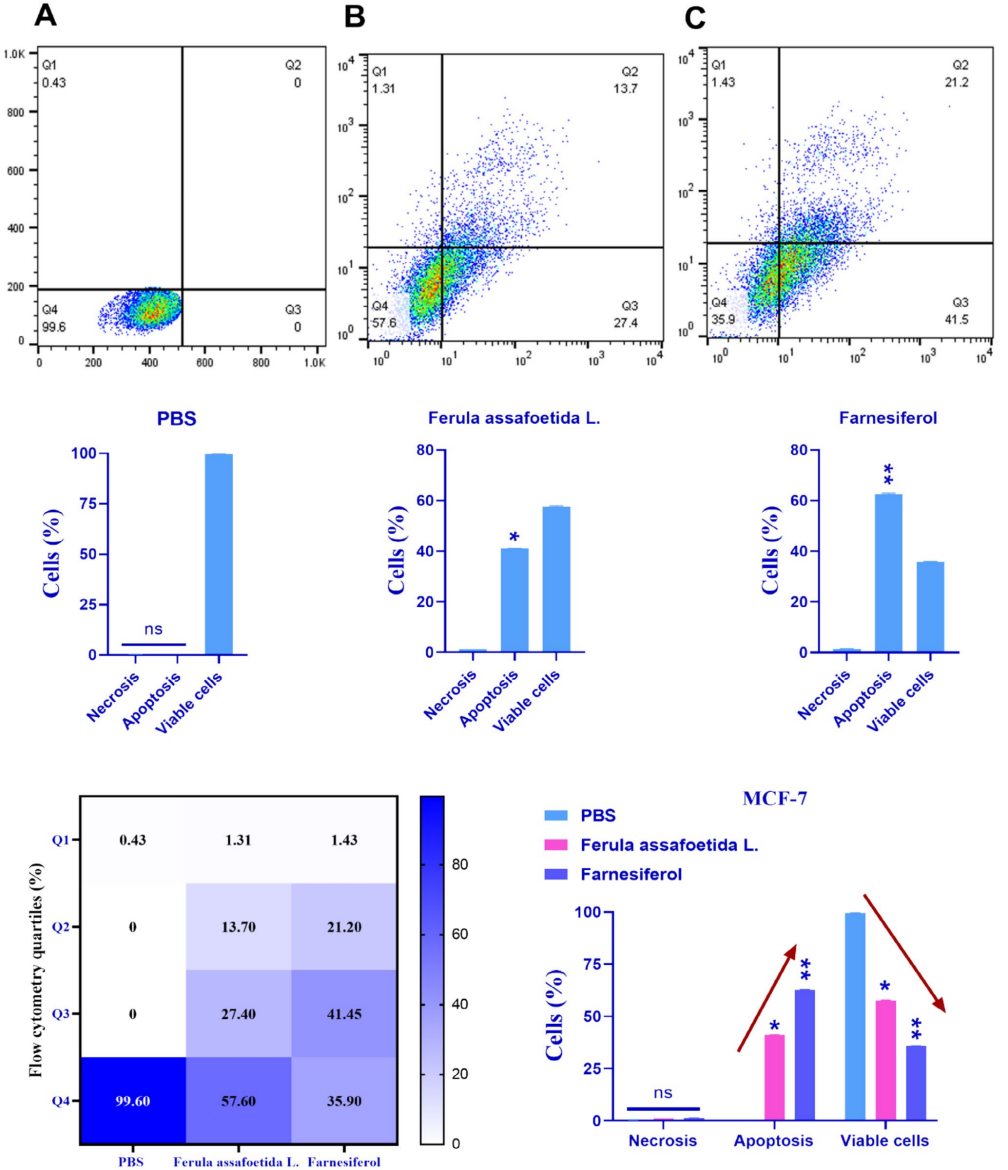
In MCF‐7 cells subjected to PBS (A), *Ferula assafoetida* L. (B), and *Farnesiferol* (C), necrotic and apoptotic cells are identified using a flow cytometer. Q1: Percentage of cells experiencing necrosis; Q2: Proportion of cells in the early stages of apoptosis; Q3: Proportion of cells in the late phases of apoptosis; Q4: Proportion of viable cells. The data were expressed as the mean value ± standard deviation (SD). An ANOVA test was used for this investigation. *p < 0.05; **p < 0.01.

The MCF‐7 and MDA‐MB‐231 cell lines were treated with PBS, *Ferula assafoetida* L., and *farnesiferol* for 72 h, and the stimulation of apoptosis was assessed using the Annexin‐V‐FITC assay. The Annexin‐V preparations include Annexin‐V for evaluating membrane asymmetry and a viability dye for assessing membrane integrity. Phosphatidylserine (PS), a negatively charged phospholipid found on the inner side of the plasma membrane, appears on the outer cell surface during the first phase of apoptosis. The test indicates that the Q1 region comprises necrotic cells, the Q2 region includes cells in the late phase of apoptosis, the Q3 region consists of cells in the early phase of apoptosis, and the Q4 region contains viable cells. The findings indicated that treatment with *Ferula assafoetida* L. and *Farnesiferol* prompted apoptosis in MCF‐7 cells at rates of around 41.1% and 62.7%, respectively (Figure 2). The apoptotic percentage in the *Farnesiferol* group was considerably elevated compared to the control group (PBS). Furthermore, 21.2% of MCF‐7 cells exhibited secondary apoptosis and 1.43% necrosis after treatment with the IC_50_ dosage of *Farnesiferol* after 72 h. The early apoptosis and survival rate within this cohort have been determined to be 41.5% and 35.9%.

**FIGURE 3:**
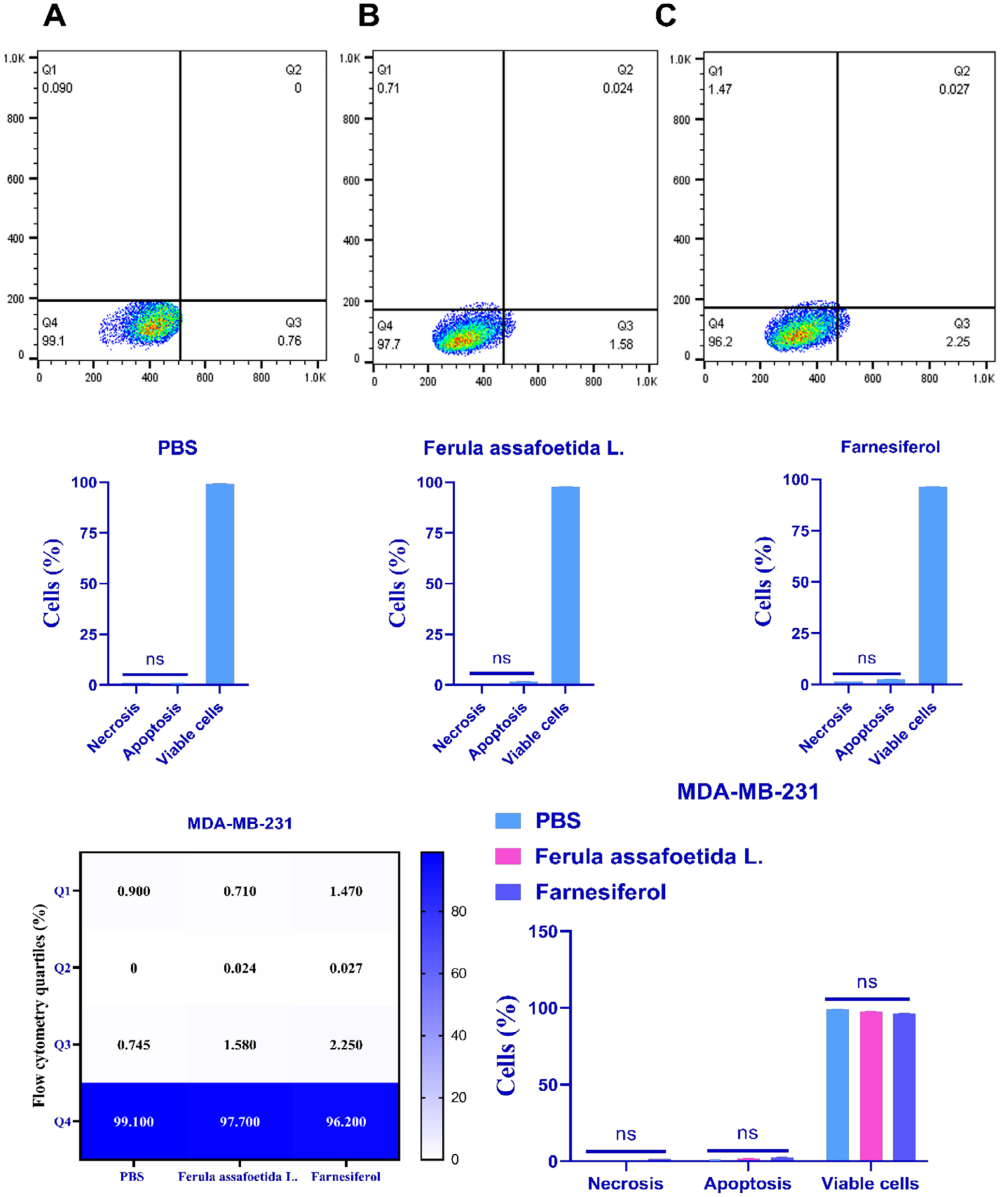
In MDA‐MB‐231 cells subjected to PBS (A), *Ferula assafoetida* L. (B), and *Farnesiferol* (C), necrotic and apoptotic cells are identified using a flow cytometer. Q1: Percentage of cells experiencing necrosis; Q2: Proportion of cells in the early stages of apoptosis; Q3: Proportion of cells in the late phases of apoptosis; Q4: Proportion of viable cells. The data were expressed as the mean value ± standard deviation (SD). An ANOVA test was used for this investigation. *p < 0.05; **p < 0.01.

Treatment of MDA‐MB‐231 cells with *Ferula assafoetida* L. and *farnesiferol* yielded apoptotic rates of around 1.60% and 2.27%, respectively (Figure 3). The *farnesiferol* therapy resulted in a marginally elevated apoptotic rate, which was not statistically distinct from the control group (PBS). A subsequent study after 72 h of treatment with the IC_50_ dosage of *farnesiferol* indicated that 0.027% of cells had secondary apoptosis, whereas 1.47% experienced necrosis. The early apoptotic rate in this cohort was 2.25%, accompanied by a survival rate of 962%. The apoptotic rate induced by *farnesiferol* therapy did not considerably surpass that of the *Ferula assafoetida* L. group (Figure 3).

### 3.3 Investigation of Cell Cycle Arrest

**FIGURE 4:**
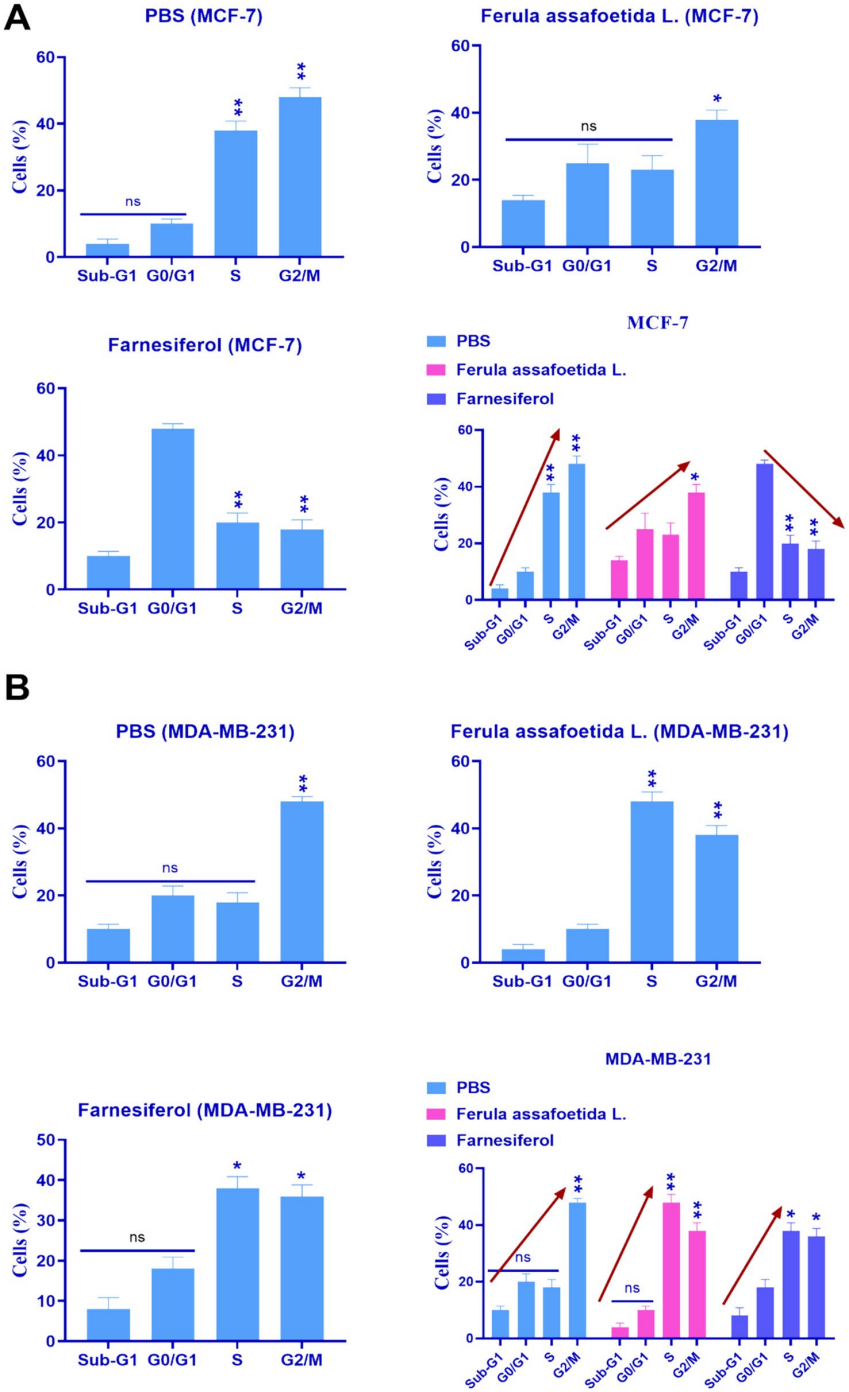
The progression of the cell cycle in (A) MCF‐7 and (B) MDA‐MB‐231 cells was analyzed in response to several treatments: PBS, *Ferula assafoetida* L., and *farnesiferol*. Statistically significant differences were noted: *p < 0.05 denotes a significant difference, whereas **p < 0.01 signifies a very significant difference.

The alteration in the cell cycle after 72‐h treatment of the MCF‐7 and MDA‐MB‐231 cell lines with PBS, *Ferula assafoetida* L., and *farnesiferol* was examined by flow cytometry. According to Figure 4A, *farnesiferol* prevented the MCF‐7 breast cancer cell line from entering the G0/G1 phase. This study's findings also validated the rise in apoptosis in the MCF‐7 breast cancer cells. The cell cycle analysis indicated that MCF‐7 cells in the *farnesiferol* group had the lowest percentage of progression into the G2/M phase (*p* < 0.05*). Analysis indicated that MDA‐MB‐231 breast cancer cells did not demonstrate growth arrest throughout the early (G0/G1) or DNA synthesis (S) stages of the cell cycle (Figure 4B). No indication of programmed cell death (apoptosis) was seen in MDA‐MB‐231 cells. Cell cycle research revealed that MDA‐MB‐231 cells had a markedly increased tendency to progress into the G2/M phase when cell division transpires (*p* < 0.05) (Figure 4B).

### 3.4 *Farnesiferol* Increases Transcription of Invasiveness Genes

**FIGURE 5:**
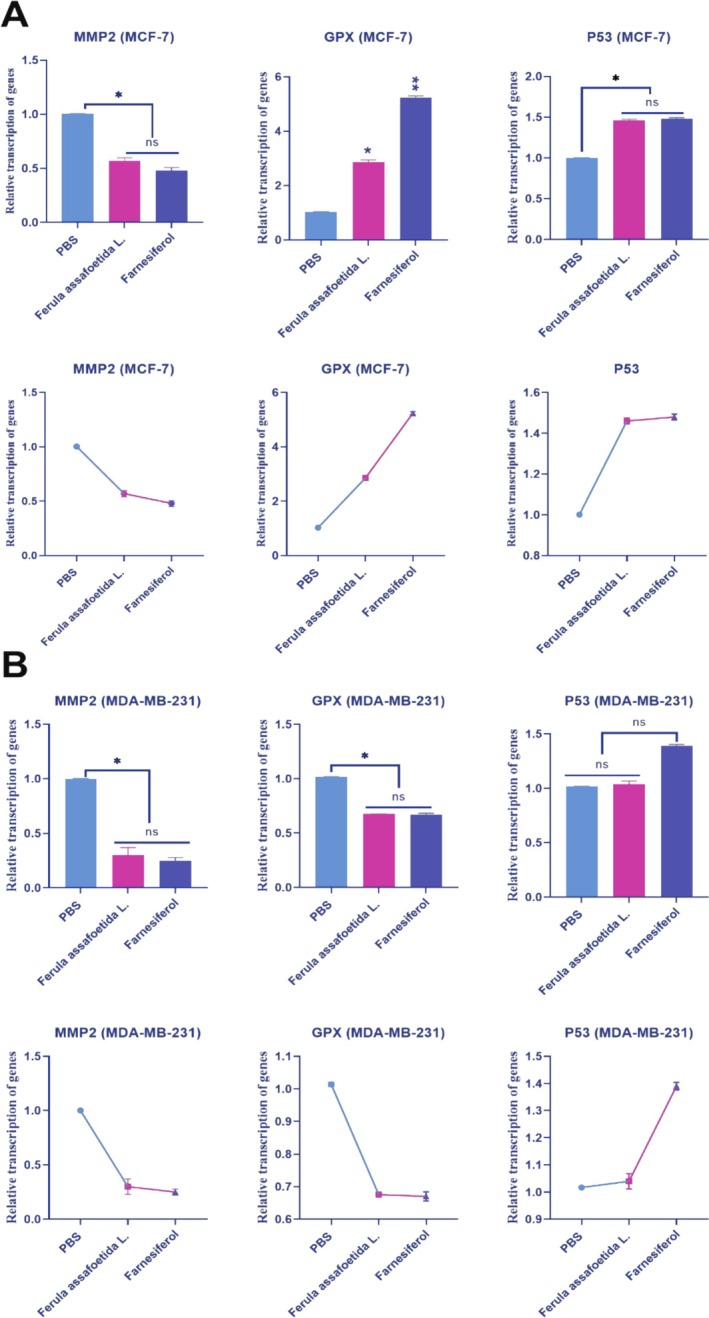
Real‐time PCR results in (A) MCF‐7 and (B) MDA‐MB‐231 cells treated with PBS, *Ferula assafoetida* L., and *farnesiferol*. The transcription of the *GPX* and *P53* genes was markedly elevated in MCF‐7 cells due to the action of *farnesiferol* compared to the control group. However, it exhibited variability in MDA‐MB‐231 cells. *p < 0.01, **p < 0.05, ns, Not significant.

We examined the impact of PBS, *Ferula assafoetida* L., and *farnesiferol* on gene transcription in MCF‐7 and MDA‐MB‐231 cells. This study established a relationship among PBS, *Ferula assafoetida* L., and *farnesiferol*, the activation of *GPX* and *P53* genes, and the suppression of the *MMP2* gene in MCF‐7 cells. The transcription rates of the *GPX* and *P53* genes were significantly increased in MCF‐7 cells treated with *Ferula assafoetida* L. and *farnesiferol* compared to the PBS‐treated group. The transcription levels of the *GPX* gene in the *Ferula assafoetida* L. and *farnesiferol* groups were 2.86 and 5.24, respectively. In contrast, the transcription rate in the PBS control was 1.026. *Ferula assafoetida* L. and *farnesiferol* groups had *P53* gene transcription levels of 1.46 and 1.48, respectively. On the other hand, the PBS control had a transcription level of 1.0013. In MCF‐7 cells treated with *Ferula assafoetida* L. and *farnesiferol*, the transcription rate of the *MMP2* gene was significantly lower than in the PBS‐treated group. *Ferula assafoetida* L. and *farnesiferol* groups had *MMP2* gene transcription scores of 0.57 and 0.48, respectively. In contrast, the PBS control had a transcription level of 1.0041. The findings demonstrated a significant 1‐fold to 2‐fold elevation in the transcription levels of the *GPX* and *P53* genes, associated with programmed cell death, after administration with *farnesiferol* (**p < 0.01) (Figure 5A).

The transcription of the *GPX* and *P53* genes exhibited variability in response to *farnesiferol* in MDA‐MB‐231 cells. *Ferula assafoetida* L. and *farnesiferol* groups had *GPX* gene transcription scores of 0.675 and 0.670, respectively. The transcription ratio in the PBS control was 1.014. The *P53* gene transcription values for *Ferula assafoetida* L. and *farnesiferol* groups were 1.04 and 1.39, respectively. Conversely, the PBS control had a transcription score of 1.017. In MDA‐MB‐231 cells subjected to *Ferula assafoetida* L. and *farnesiferol* treatment, the transcriptional activity of the *MMP2* gene was markedly reduced compared to the PBS‐treated group. The transcription scores for the *MMP2* gene were 0.30 for *Ferula assafoetida* L. and 0.25 for the *farnesiferol* groups. The PBS control had a transcription score of 1.0014. The results indicated that the transcription of *GPX* and *P53* genes varied in response to *farnesiferol* in MDA‐MB‐231 cells (Figure 5B).

### 3.5 Total Antioxidant Capacity (TAC) Results

**FIGURE 6:**
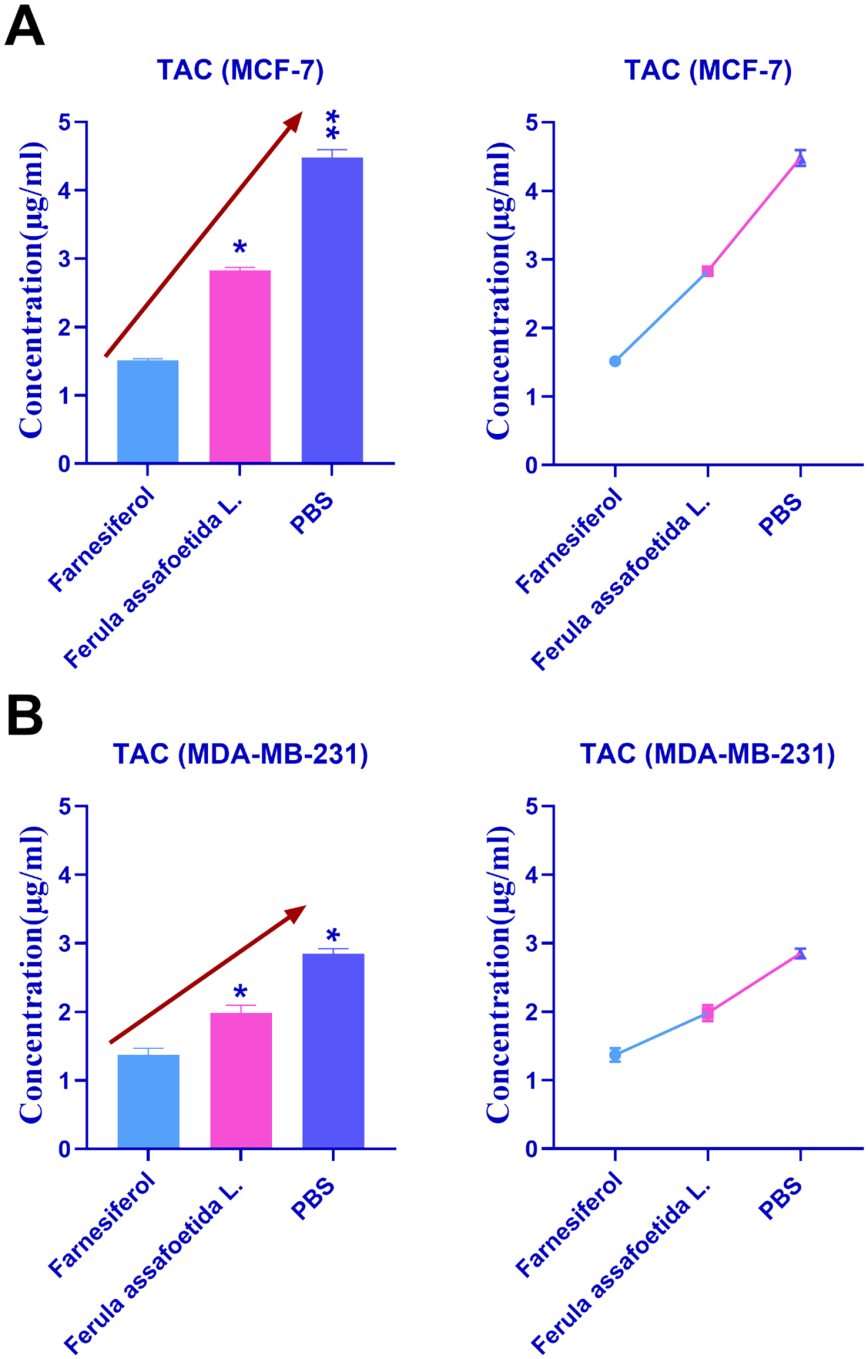
The antioxidant properties in MCF‐7 (A) and MDA‐MB‐231 (B) cells treated with *Ferula assafoetida* L. and *farnesiferol* exhibited a decreased total antioxidant capacity relative to the PBS control group. Statistically significant differences were noted: * *p* < 0.05 denotes a significant difference, whereas ** *p* < 0.01 signifies a very significant difference.

Figure 6 depicts the comparative results of antioxidant characteristics in MCF‐7 and MDA‐MB‐231 cells treated with PBS, *Ferula assafoetida* L., and *farnesiferol*. Figure 6A demonstrates that treatment with *Ferula assafoetida* L. and *farnesiferol* in MCF‐7 cells decreased the total antioxidant capacity compared to the PBS control group. The TAC of *Ferula assafoetida* L. and *PBS* in MCF‐7 cells was 2.83 and 4.48, respectively. Figure 6B demonstrates that treatment with *Ferula assafoetida* L. and *farnesiferol* in MDA‐MB‐231 cells decreased the total antioxidant capacity compared to the PBS control group. The TAC of *Ferula assafoetida* L. and *PBS* in MDA‐MB‐231 cells was 1.98 and 2.85, respectively. The augmentation of antioxidant activities by the active component *farnesiferol* significantly surpassed those of *Ferula assafoetida* L.

## 4 Discussion

Chemotherapy, a systemic intervention for breast cancer, employs pharmacological agents to eradicate quickly proliferating cells, including cancerous cells (National Cancer Institute 2025). It can be administered preoperatively to reduce tumor size for simpler excision or postoperatively to eliminate residual cancer cells and diminish the likelihood of recurrence (National Cancer Institute 2025). Chemotherapy is an essential element of breast cancer treatment protocols, offering substantial therapeutic advantages, including enhanced likelihood of remission, improved long‐term survival rates, and a possible decrease in the need for extensive surgical interventions (Early Breast Cancer Trialists' Collaborative Group (EBCTCG) 2005). Although contemporary chemotherapy has improved its precision in targeting cancer cells, it nevertheless unavoidably has undesirable side effects. This underscores the pressing need for continuous investigation into innovative cancer therapies (Ozols 2000). For millennia, plant‐derived chemicals have been acknowledged as a significant and promising resource for the discovery of novel anticancer therapeutics (Newman et al. 2003).

*Ferula assafoetida* L., commonly known as asafoetida, is a perennial plant indigenous to Iran and Afghanistan (Iranshahi et al. 2009). It comprises an intricate amalgamation of chemicals, including sulfide compounds, flavonoids, and terpene coumarins (Kashiwada et al. 1990). Initial studies indicate that certain chemicals generated from asafoetida may have anticancer effects. These chemicals have shown the ability to impede the development of several cancer cell lines in laboratory tests by causing apoptosis and suppressing cellular proliferation. Further investigation is required to comprehensively elucidate the mechanisms of action and to establish the safety and effectiveness of asafoetida‐derived compounds in the treatment of human cancer (Iranshahi et al. 2009; Kashiwada et al. 1990; Lee et al. 2011). Farnesiferol, a chemical extracted from *Ferula assafoetida* L., has shown potential anticancer effects in initial studies (Lee et al. 2011).

Research indicates that farnesiferol may impede the proliferation of several cancer cell types by triggering apoptosis and inhibiting cellular growth (Mousavi et al. 2013). Studies have examined the possible mechanisms of action of farnesiferol, indicating that it may influence particular signaling pathways associated with cancer formation and progression (Rahgozar et al. 2020). It is essential to acknowledge that these data are mostly derived from in vitro (cell culture) and in vivo (animal) investigations (Mousavi et al. 2013; Rahgozar et al. 2020). Additional study is required to comprehensively assess its therapeutic properties, including safety and effectiveness in humans, prior to its consideration for clinical use in cancer therapy (Abdolahi et al. 2019).

Farnesiferol treatment may significantly reduce total antioxidant capacity (TAC). Our investigations on MDA‐MB‐231 cells revealed a total antioxidant capacity (TAC) of 1.98 for farnesiferol and a significantly elevated TAC of 2.85 for *Ferula assafoetida* L. extract. These results underscore the diminished antioxidant properties of the active compound farnesiferol in comparison to the whole plant extract (Abdolahi et al. 2019; Re et al. 1999). Research indicates that farnesiferol has antioxidant characteristics, potentially affecting the total antioxidant activity of cells or tissues (Mousavi et al. 2012). Our results in MCF‐7 and MDA‐MB‐231 cells corroborate this fact, revealing a markedly lower TAC in cells treated with farnesiferol relative to those treated with the entire plant extract of *Ferula assafoetida* L. This finding diverges from the general understanding that isolated bioactive compounds often exhibit more potent and targeted effects compared to crude extracts due to increased purity and concentration (Pan et al. 2013). For instance, studies on other plant‐derived compounds, such as curcumin, have also shown that purified forms can offer superior antioxidant benefits than their parent extracts (Pan et al. 2013; Cragg and Pezzuto 2016; Li et al. 2019; Newman and Cragg 2016).

In this investigation, we used farnesiferol, a chemical extracted from the medicinal plant *Ferula assafoetida* L., to investigate the possibility of an anti‐apoptotic impact on breast cancer cells. Apoptosis, or programmed cell death, is an essential process for the removal of damaged or undesirable cells, including neoplastic cells (Newman and Cragg 2016; National Cancer Institute 2025; Early Breast Cancer Trialists' Collaborative Group (EBCTCG) 2005; Ozols 2000; Newman et al. 2003; Iranshahi et al. 2009; Kashiwada et al. 1990; Lee et al. 2011; Mousavi et al. 2013; Rahgozar et al. 2020; Abdolahi et al. 2019; Re et al. 1999; Mousavi et al. 2012; Elmore 2007). We propose that farnesiferol may promote apoptosis in breast cancer cells, hence enhancing its potential anticancer efficacy.

Our results indicated that farnesiferol therapy markedly enhanced apoptosis in cancer cell lines. This discovery was corroborated by other lines of evidence, including an upregulation of pro‐apoptotic genes and a downregulation of anti‐apoptotic genes (Lee et al. 2011; Mousavi et al. 2012; Zhang et al. 2019). The study indicates that farnesiferol may partially exert its anticancer actions by inducing apoptosis in breast cancer cells, hence encouraging their demise. Studies indicate that farnesiferol therapy may trigger apoptosis in cancer cell lines. Research has shown this impact across several cancer types, indicating its potential as a promising anticancer drug (Lee et al. 2011; Mousavi et al. 2012; Zhang et al. 2019).

Our investigation revealed encouraging results with enhanced apoptosis in MCF‐7 breast cancer cells after farnesiferol therapy; however, we observed conflicting results in MDA‐MB‐231 cells. This disparity underscores the intricate and diverse nature of breast cancer, highlighting the need to account for the distinct attributes of various cell types. A potential reason for this gap resides in the inherent distinctions between MCF‐7 and MDA‐MB‐231 cells. MCF‐7 cells are estrogen receptor‐positive (ER+), but MDA‐MB‐231 cells are estrogen receptor‐negative and triple‐negative, missing estrogen receptor, progesterone receptor, and human epidermal growth factor receptor 2 (Hollestelle et al. 2013; Kavousi and Chavoshi 2020). These unique molecular profiles may markedly affect cellular responses to diverse therapies, including farnesiferol. This differential response is a significant finding that necessitates further exploration. Previous research on other natural compounds has also reported varied apoptotic effects across different breast cancer subtypes. The reduced apoptotic effect of farnesiferol in MDA‐MB‐231 cells suggests that its mechanism of action might be more effective in hormone receptor‐positive contexts or that TNBC cells possess inherent resistance mechanisms that need to be overcome. Additional inquiry is essential to clarify the underlying processes responsible for these divergent results (Dai et al. 2018; Piri‐Gharaghie et al. 2022). This may include investigating differential gene expression patterns, analyzing changes in cellular signaling pathways specifically in the context of receptor status, and evaluating the effects of farnesiferol on certain biological processes unique to these two cell lines.

## 5 Conclusion

Our research indicates that farnesiferol, a chemical derived from *Ferula assafoetida* L., has significant anticancer potential. We observed a substantial reduction in TAC with farnesiferol treatment, while *Ferula assafoetida* L. extract showed a higher TAC. Additionally, farnesiferol treatment led to a notable induction of apoptosis in breast cancer cells. The data suggest that farnesiferol may provide a new treatment strategy for breast cancer. The observed differential response in MCF‐7 and MDA‐MB‐231 cells highlights the importance of breast cancer subtype in therapeutic efficacy and points to the need for tailored approaches. Further study is essential to comprehensively clarify the underlying mechanisms of action, including its impact on particular signaling pathways and interactions with other anticancer medicines, especially in triple‐negative breast cancer. Extensive preclinical and clinical investigations are necessary to assess the safety and effectiveness of farnesiferol as a prospective therapeutic agent for breast cancer.

## Author Contributions

**Farinaz Malakotikhah:** investigation (equal), methodology (equal), validation (equal), writing – original draft (equal), writing – review and editing (equal). **Kahin Shahanipour:** investigation (equal), methodology (equal), software (equal), validation (equal), writing – original draft (equal), writing – review and editing (equal). **Ramesh Monajemi:** formal analysis (equal), investigation (equal), methodology (equal), software (equal), validation (equal), writing – original draft (equal), writing – review and editing (equal). **Ali Mohammad Ahadi:** investigation (equal), methodology (equal), resources (equal), software (equal), validation (equal), writing – original draft (equal), writing – review and editing (equal). **Ali Asghar Rastegari:** formal analysis (equal), investigation (equal), methodology (equal), software (equal), validation (equal), writing – original draft (equal), writing – review and editing (equal).

## Consent

The authors have nothing to report.

## Conflicts of Interest

The authors declare no conflicts of interest.

## Data Availability

The datasets analyzed during the current study are available from the corresponding author upon reasonable request.
